# Effect of screen time on behavior of pre-schoolers in Islamabad

**DOI:** 10.12669/pjms.39.2.6883

**Published:** 2023

**Authors:** Marriam Suleman, Ume Sughra, Asmaa Riaz, Maheen Akbar

**Affiliations:** 1 Dr. Marriam Suleman, MBBS, MSPH., Al-Shifa Research Centre, Al-Shifa Trust Eye Hospital Main GT Road, Near Ayub Park, Rawalpindi, Pakistan; 2 Prof. Dr. Ume Sughra, MBBS, MPH, FCPS, FRCP, MCPS-HPE., Al-Shifa Research Centre, Al-Shifa Trust Eye Hospital Main GT Road, Near Ayub Park, Rawalpindi, Pakistan; 3 Dr. Asmaa Riaz, BDS MSPH., Al-Shifa Research Centre, Al-Shifa Trust Eye Hospital Main GT Road, Near Ayub Park, Rawalpindi, Pakistan; 4 Dr. Maheen Akbar, MBBS FCPS, FRCP., Al-Shifa Research Centre, Al-Shifa Trust Eye Hospital Main GT Road, Near Ayub Park, Rawalpindi, Pakistan

**Keywords:** Pre-schoolers, Screen Time, Withdrawal Syndrome, Autism Spectrum Problems, Child behavior Checklist

## Abstract

**Objective:**

Early years of childhood form the basis of intelligence, personality, social behaviour, and capacity to learn and nurture oneself as an adult. Our objective was to find out the effects of screen time on behavior of pre-schoolers, which could provide scientific grounds to the control of digital screen time.

**Method:**

A cross sectional survey was conducted in four private preschools of Islamabad from June -November 2021. A sample size of 200 children ages three-five years were selected through multistage random sampling using a parental questionnaire. Children were grouped based on daily screen time of ≤60 minutes or >60 minutes. Analysis was made based on the Child behavior checklist for ages eighteen months-five years results. Cronbach’s alpha coefficient was found to be 0.925. It was analyzed using SPSS version 22. Chi-square test, independent sample t-test and multi linear regression were applied to determine the association and significance levels between the variables.

**Results:**

Study results indicate increased screen time was statistically significant with child’s age, education level and employment status of mothers. It was observed that pre-schoolers with screen time of > 60 minutes tend to suffer more from withdrawal syndrome (11.94±3.91, p = 0.014) sleep problems (10.97±3.20, p = 0.010) and Autism spectrum problems (17.66±5.89, p = 0.047) as compared to pre-schoolers with screen time ≤60 minutes. Strongest predictor of outcome variable was found to be mothers education level (ß = 21.53).

**Conclusion:**

Study findings revealed that excessive screen time has deleterious effect and is associated with behavioural problems of pre-schoolers.

## INTRODUCTION

Electronic devices have become an integral part of our daily life. Screen-time is the duration of time spent by individual in using electronic/digital media like television, smartphone, tablet, or computer.^1^ Age of a pre-schoolers is defined as three-five years by centers for Disease Control and Prevention.^2^ It is recommended by American Academy of Paediatrics to limit screen time for children aged two-five years to one hour/day.^3^ Children’s screen time is increasing, and they start using it in earlier years of life.

These new trends are concerning because excessive screen time in early childhood is associated with adverse physical, psychosocial, and cognitive outcomes.^4^ Evidence shows that behavior formed during the preschool years are stable and can be tracked into late childhood.^5^ Research findings link high screen time with irritability, negative mood, cognitive and socioemotional development, consequently leading to poor educational performance. Another negative impact is on sleep quality.^6^

**Fig.1:**
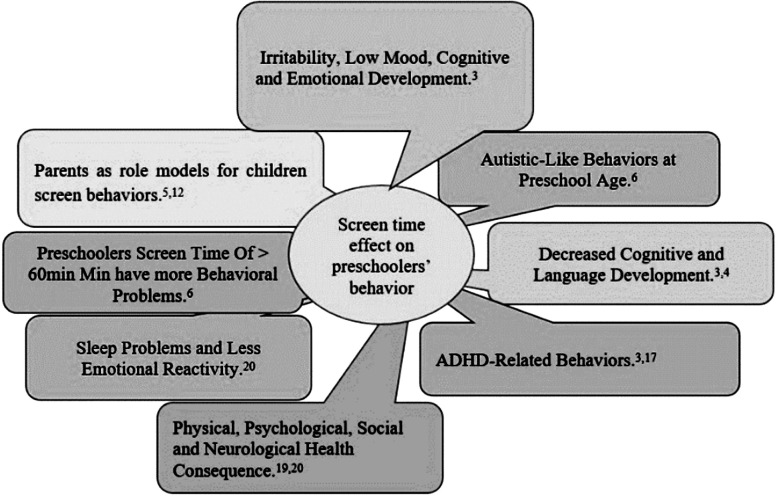
Conceptual Map of current study outcome variable based on literature review.

In a recent systematic review, moderately strong evidence was found between screen time and depressive symptoms and weak associations were found between screen time and behavior problems, anxiety, hyperactivity, inattention, and poor sleep.^7^ A survey conducted on 1200 families in U.S, found out 45% children preferred indoor activities like watching screens or playing games on devices (43%) as compared to indoor screen-free activity like playing with a sibling/friend (26%) or playing alone (16%)^8^ (Fig.1).

Studies are more focused on the effects of addictive use of digital media on schoolers, but rarely on pre-schoolers.^6^ The major problem regarding this concern is that worldwide research has been conducted in this regard especially in last five years but unfortunately very few studies have been done so far in Pakistan and the topic has not been explored in detail. Therefore, studying this issue is important to assess the effect of screen time on behavior of pre-schoolers in Islamabad.

This research is significant in finding the effects of screen time on behavior of pre-schoolers. This research will further motivate and provide guidelines for parents and for kindergarten teachers as well to help decrease pre-schoolers screen time and engage them in other healthy activities.

## METHODS

It was a descriptive cross-sectional study conducted in four private preschools of Islamabad from June -November 2021 with a sample size of 200 calculated by using formula N=z^2^ (p × q)/e^2^. Data was collected through multistage random sampling equally divided with respect to gender and schools (50 each) from private preschools of Islamabad.

The independent variable of the study was screen time of pre-schoolers. The tool used to assess screen time of pre-schoolers was SCREENS-Q.^9^ It is a parent-reported standardized comprehensive questionnaire tool to assess children’s screen media habits at home.

The questionnaire used to assess behavior of pre-schoolers was adapted from CHILD BEHAVIOR CHECKLIST FOR AGES 1½-5 (CBCL/1.5-5), consisting of 99 items assessed on three - point Likert scale.^10^ Children’s behavior was divided into eight categories, including Emotionally Reactive, Anxious/Depressed, Aggressive Behavior, Attention Problems, Somatic Complaints, Withdrawal Symptom, Sleep Problems and Other Problems. The outcome variable of study was behavior of pre-schoolers which was kept quantitative. The socio demographic variables taken were all qualitative in nature, which included age, gender, education level of children and parents, primary care giver and employment status of parents. Cronbach’s alpha was found to be 0.925. Data entry and statistical analysis were done using SPSS software version 22.

The items forming the outcome variables were reported in mean and standard deviation. Socio-demographic characteristics and screen time were analyzed using chi-square analysis. To compare screen time and CBCL Independent samples t-test was used with a p-value less than or equal to 0.05 was considered as statistically significant. Multiple linear regression was done to check for predictors.

The study was approved by Ethical Review Committee of Pakistan Institute of Ophthalmology, Al-Shifa Trust Eye Hospital Rawalpindi (Reference No: ERC-11/AST -21) dated 1^st^ June 2021. Informed consent was obtained from the parents/ guardians of the respondents in written form.

## RESULTS

Out of 200 participants, 88(44.0%) were five years old. Among the participants 100 (50%) were male and 100(50%) were females. Regarding the education level of the children, most of the pre-schoolers were studying in early years one, 83 (41.5%). Education level of fathers showed, 188(94%) had completed high school or higher education. Similarly, education level of mothers had shown 188(94%) had completed high school or higher. 186(93%) parents were primary care givers, employment status of fathers had shown that 193(96.5%) were employed, 118(59.0%).

For independent variable, screen time of pre-schoolers, the number of screen media devices present in the household, 89(44.5%) had one laptop and had 80(40%) one tablet. Two smart phones were present in 89(44.5%) of participants household. 102(51.0%) had one television. Within the past two months, 62(31%) had used tablet and 108(54%) had used smart phone every day, 119(59.5%) had watched television every day. Devices owned by the participants were 33(16.5%) had smart phones, 36(18%) of participants had television. 70(35%) of participants had used screen media devices daily, in connection with preschool related activities.

On weekdays, within the past two months, 59(29.5%) pre-schoolers had spent 30-59 minutes watching entertainment programs, 44(22.0%) had spent 30-59 minutes playing games on screen media devices, 57(28.5%) had spent 1-29 minutes on preschool related activities. On weekend days, within the past two months, 54(27.0%) pre-schoolers had spent 30-59 minutes watching entertainment programs, 47(23.5%) had spent 1-29 minutes playing games on screen media devices, 49(24.5%) had spent 1-29 minutes on preschool related task. On weekdays, within the past two months, 54(27.0%) parents had spent 30-59 minutes on entertainment programs, 1- 29 minutes were spent on video calls by 58(29.0%) parents, surfing web was 1-29 minutes by 53(26.5%). On weekend days, within the past two months, 57(28.5%) parents had spent 30-59 minutes on entertainment programs, 53(26.5%) had spent 30-59 minutes on social media and surfing web was 1-29 minutes by 27.0% (n=54) parents.

Within the past two months, 159 (79.5 %) parents of pre-schoolers had spent less than and equal to 60 minutes and 41(20.5%) had spent more than 60 minutes on screen media devices per day. Among CBCL, the highest score of pre-schoolers was of Other Problems 43.47 ± 10.796. The lowest score was of Attention Problem 7.73 ± 2.110. Internalizing Problems score was 49.50 ± 12.515, higher than externalizing problems. Among DSM-Oriented Scales, highest score was of Autism Spectrum Problems, 16.67 ± 5.177.

For inferential results, Chi-square test of independence was performed to determine the association between socio- demographic characteristics and screen time of pre-schoolers as both variables are qualitative categorical. Screen time was categorized into ≤60 minutes and >60 minutes. (Table-I). Chi-square test of independence was performed to determine the association between Screen time of pre-schoolers and screen time of parents. According to the results, there was statistically significant association between these two variables. P = 0.001. 109(54.5%) children and parents screen time were ≤60 minutes as compared to 29(14.5%) children and parent screen time was >60 minutes.

**Table-I:** Socio-Demographic Characteristics of Study Population

Variables	Screen Time n (%)		Total (n=200)	Chi-q Value	p Value

	≤60min	>60min			
***Age of child***				12.725	0.002
3 years	42(21.0%)	17(8.5%)	59(29.5%)		
4 years	38(19.0%)	15(7.5%)	53(26.5%)		
5 years	41(20.5%)	47(23.5%)	88(44.0%)		
Gender of child				0.188	0.664
Female	59(29.5%)	41(20.5%)	100(50%)		
Male	62(31.0%)	38(19.0%)	100(50%)		
***Father education level***				0.948	0.623
High school or higher	115(57.5%)	73(36.5%)	188(94.0%)		
Secondary school	4(2.0%)	3(1.5%)	7(3.5%)		
Primary school or lower	2(1.0%)	3(1.5%)	5(2.5%)		
***Mother education level***				0.482	0.786
High school or higher	113(56.5%)	75(37.5%)	188(94.0%)		
Secondary school	7(3.5%)	3(1.5%)	10(5.0%)		
Primary school or lower	1(0.5%)	1(0.5%)	2(1.0%)		
***Education level of child***				6.654	0.036
Early years 1	59(29.5%)	24(12.0%)	83(41.5%)		
Early years 2	29(14.5%)	26(13.0%)	55(27.5%)		
Early years 3	33(16.5%)	29(14.5%)	62(31.0%)		
***Father Employment status***				1.930	0.165
Employed	115(57.5%)	78(39.0%)	193(96.5%)		
Unemployed	6(3.0%)	1(0.5%)	7(3.5%)		
***Mother Employment status***				5.009	0.025
Employed	42(21.0%)	40(20.0%)	82(41.0%)		
Unemployed	79(39.5%)	39(19.5%)	118(59.0%)		
Primary care giver				2.854	0.240
Parents	110(55.0%)	76(38.0%)	186(93.0%)		
Grand parents	10(5.0%)	2(1.0%)	12(6.0%)		
Nany	1(0.5%)	1(0.5%)	2(1.0%)		

Independent sample t-test was run to check the Mean score comparisons between children categorized by screen time daily on CBCL. To study the effects of screen time on children’s behaviours, the participants were divided into two groups based on screen time of less than or over 60 minutes per day. (Table-II)

**Table-II:** Mean Score Comparisons between Children Categorized by Screen Time Daily on CBCL

Syndromes scales	Screen time		t (df)	P-Value

	≤60min mean ±SD	> 60 min mean ±SD		
Internalizing	48.26±10.82	51.39±14.61	1.73(198)	0.084
Emotionally reactive	12.22±3.27	13.13±4.46	1.54(131.77)	0.124
Anxious/depressed	11.21±2.62	11.70±3.45	1.11(198)	0.266
Somatic complaints	14.11±3.28	14.63±4.25	0.98(198)	0.327
Withdrawn	10.72±3.01	11.94±3.91	2.48(198)	0.014
Sleep problems	9.90±2.59	10.97±3.20	2.605(198)	0.010
Externalizing	35.79±9.05	37.65±10.91	1.308(198)	0.192
Attention problems	7.06±2.05	7.91±2.19	1.01(198)	0.314
Aggressive behaviour	28.18±7.45	29.73±9.18	1.312(198)	0.191
Other problems	42.68±9.87	44.67±12.03	1.27(198)	0.203
Total problems	136.63±30.77	144.68±39.19	1.622(198)	0.106
DSM- oriented scales	62.92±14.51	66.90±18.47	1.700(198)	0.91
Depressive problems	13.30±3.33	14.32±4.32	1.874(198)	0.062
Anxiety problems	13.96±3.13	14.85±4.36	1.676(198)	0.095
Autism spectrum problems	16.17±4.58	17.66±5.89	1.997(198)	0.047
Attention deficit/hyperactivity problems	10.44±2.77	10.87±3.07	1.038(198)	0.300
Oppositional defiant problems	9.05(2.85)	9.20(3.32)	0.347(198)	0.729

Multiple linear regression conducted to find out predictors for the outcome variable. Socio demographic characteristics, screen time of child and parents were checked during preliminary analysis. (Table-III)

**Table-III:** Multiple Linear Regression Model

R²	Adjusted R²	Standard Error	F	p-value	

0.087	0.038	33.811	1.795	0.064	

Variables	Unstandardized β	t	p-value	95% CI	

				Lower	Upper
Constant	105.33	4.359	0.001	57.66	153.003
Age of child	5.145	1.35	0.177	2.33	12.629
Gender of child	5.495	1.117	0.265	4.20	15.196
Education level of child	0.851	0.228	0.820	8.19	6.497
Father education level	5.587	0.749	0.455	20.30	9.135
Mother education level	21.531	2.44	0.015	4.15	38.90
Primary care giver	9.779	1.196	0.233	25.91	6.35
Father employment status	2.529	0.182	0.856	29.90	24.84
Mother employment status	4.436	0.852	0.395	14.70	5.83
Screen time child	1.795	0.32	0.744	9.04	12.63
Screen time parents	14.575	2.28	0.024	1.96	27.18

## DISCUSSION

This research was aimed to study the effect of screen time on behavior of pre-schoolers and to provide guidelines for pre-schoolers screen time. The observation related to age of child that is five years old having screen time > 60 minutes (n=47,23.5%, p = 0.002) agrees with previous study that as the child age increases so does their screen time.^11^ Similarly, as education level of pre-schoolers is having significant association with screen time (n=29,14.5%, p = 0.036). Another finding of present study was maternal education level, that was found to be the strongest predictor (ß = 21.53). Maternal education is also found to be significant in another study conducted in Greece on pre-schoolers to determine the factors associated with television viewing.^12^

The current study has also shown significant association between screen time of pre-schoolers and employment status of mothers, (n=40, 20.0%, p=0.025). There was statistically significant association between children and parents screen time ≤60 minutes. (n=109, 54.5%, P = 0.001), another study reported that parental television viewing time is most important determinant of children television viewing time reason.^12^

This research was conducted in urban areas of Islamabad, rural areas were not included, people living in rural areas of Pakistan don’t have good access to screen media devices, due to low socioeconomic status and mainly rural areas doesn’t have any internet infrastructure. According to the Global Information Society Watch, internet usage in rural areas of Pakistan is likely to be less than eight percent.^13^

In current research, those pre-schoolers were included who had an access and were inclined towards watching screen media devices. Another study reported that lower socioeconomic households were consistently meeting the screen time guidelines at each time point.^14^ Pre-schoolers studying in public schools were found to have limited access to screen media devices. This was the reason public preschools were not included in the current study.^15^

Present study identifies that exposing preschooler to screen time >60 minutes increases the risk of developing withdrawn syndrome (11.94±3.91, p=0.014) and autism spectrum problems (17.66±5.89, p=0.047). A study conducted at kindergartens of China,^6^ has also reported the association between exposure to screen time in early life and the presence of autistic-like behaviours among preschool children. According to another study conducted in Pakistan on school going children found significant association between long-term smartphone usage with behavioural and psychological problems.^16^

Another study has mentioned that Television is most watched screen-based device among children.^17^ This finding is similar with present research results, where television is the device that was most watched by pre-schoolers (n=119, 59.5%). Screen time is negatively associated with the development of sleep problems, depression and anxiety as mentioned in the same research.^17^ Another study conducted in Pakistan on young children, found that increased screen time is associated with aggressive behavior and anger.^18^ A rapid systematic review on psychological burden of quarantine in children and adolescents done during the COVID-19 pandemic also reported restlessness, irritability, anxiety, clinginess, and inattention with increased screen time in children during quarantine.^19^

In this research sleep quality of pre-schoolers is significantly associated with screen time >60 minutes (10.97±3.20, p=0.010). According to a randomized control study, conducted in Taiwan, when the screen time of children in experimental group was significantly reduced, they presented with improved sleep quality and attention score.^20^

### Strength:

The information this study adds to the medical literature is that Education level of pre-schoolers is having significant association with screen time. Current study has also shown significant association with mother’s employment status and screen time of pre-schoolers. This study identifies that exposing preschooler to screen time >60 minutes increases the risk of developing withdrawal syndrome. This study has provided statistically significant evidence that excessive screen time has a deleterious effect and is associated with behavioral problems of preschoolers. Clinicians when evaluating children for emotional and behavioral problems must also think of its relationship with screen time so that development and growth of children are not affected adversely.

### Limitations:

Only private preschools were included in the study, to enhance the generalizability of the findings, future studies should consider a greater number of preschools, public as well as private. Effect of screen time on language development of children could not be researched as well.

## CONCLUSION

Study findings have revealed that excessive screen time has a deleterious effect and is associated with behavioural problems of pre-schoolers. Limiting screen time at early ages is important because it helps to keep them healthy both psychologically and physically and set up good habits. Parents must also think about their child’s screen time that requires parents’ active engagement and constant attention so that development and growth of their children are not affected adversely.

### Authors Contribution:

**MS:** Conceived, protocol design, literature search, data collection, statistical analysis, interpretation of data, drafting of manuscript and is responsible for integrity of research.

**US:** Conceived, protocol design, review, and final approval of manuscript.

**AR, MA:** Data analysis, Manuscript editing and proof reading.
